# Editorial: Improving services for neglected tropical diseases: ending the years of neglect

**DOI:** 10.3389/frhs.2024.1528495

**Published:** 2024-12-02

**Authors:** Natalia Hounsome, Maya Semrau, Lawrence Rugema

**Affiliations:** ^1^ Brighton and Sussex Centre for Global Health Research, Brighton and Sussex Medical School, University of Sussex, Brighton, United Kingdom; ^2^ School of Public Health, University of Rwanda, Kigali, Rwanda

**Keywords:** neglected tropical diseases, chagas disease, mycetoma, rabies, scabies, snakebite, soil-transmitted helminths, healthcare servises

**Editorial on the Research Topic**
Improving services for neglected tropical diseases: ending the years of neglect

Neglected tropical diseases (NTDs) are a diverse group of health conditions predominantly affecting people from impoverished communities in tropical areas. They affect more than one billion people worldwide (1), mostly vulnerable population groups, and have devastating health, social and economic consequences due to disability, stigma, reduced mental wellbeing and sometimes death. The group includes bacterial infections (Buruli ulcer, leprosy, trachoma, jaws), viral infections (rabies, dengue, chikungunya), fungal infections (mycetoma, chromoblastomycosis, other deep mycoses), protozoan diseases (Chagas disease, leishmaniasis, human African trypanosomiasis), a large group of helminth diseases (lymphatic filariasis, onchocerciasis, schistosomiasis, soil-transmitted helminthiases, etc.), ectoparasitic diseases (scabies, myiasis), and non-infectious conditions (podoconiosis, snakebites).

Due to complex epidemiology and environmental factors, controlling NTDs is a challenging task. In 2012, the World Health Organization (WHO) developed the 2020 roadmap on NTDs (2), which aimed to provide populations in endemic regions with access to interventions for preventing, controlling, eliminating, and eradicating 17 neglected tropical diseases. This was followed by the WHO NTD roadmap 2021–2030, which includes 20 diseases and has a heavy focus on integration, in terms of implementing cross-cutting approaches across NTD programmes and incorporating them into national healthcare systems (3). Due to the consolidated efforts of the pharmaceutical industry, governments, non-governmental organisations, and charities, as well as expanded drug donations and financial commitments, in 2022, NTD interventions were provided to 819 million people worldwide (4). Yet, mon integration in terms of implementinging cross-cutting interventions approaches across illions of people are still living with, or are at risk of, the debilitating consequences of NTDs.

This article collection highlights the latest advancements in targeting a wide group of NTDs, including mycetoma (Ackley et al.), soil-transmitted helminths (STH) (Agrawal et al., Gwayi-Chore et al.), rabies and snakebite (Faust and Ray), scabies (Hounsome et al.) and Chagas disease (Silveira et al.). The articles address the four themes covered by the WHO 2030 targets: diagnostics (Ackley et al., Silveira et al.), monitoring and evaluation (Agrawal et al., Hounsome et al.), access and logistics (Faust and Ray), and advocacy and funding (Gwayi-Chore et al.).

The article by Ackley et al. focuses on mycetoma, a chronic, progressively destructive disease of subcutaneous tissues and bones caused by certain species of bacteria or fungi. The disease is mainly painless in the early stages. Therefore, many patients present late to healthcare facilities with advanced infection leading to tissue destruction, deformity, loss of function, limb amputation and disability. The paper presents a protocol to support the early detection and diagnosis of mycetoma in Sudan. The first stage includes exploring knowledge, attitudes, and perceptions of disease of people living in mycetoma-endemic areas to understand health-seeking behaviours, which often lead to delayed presentation to healthcare facilities. The second stage includes asset and stakeholder mapping to develop an intervention that is sustainable and uses local knowledge, skills, and resources. The protocol addresses the role of biomedicine, traditional healers, stigma, access to healthcare services and economic factors in early diagnostics of mycetoma (Ackley et al.).

The article by Silveira et al. presents the results of an interdisciplinary research project on Chagas disease in Bolivian immigrants in the city of São Paulo, Brazil. Chagas disease, or American trypanosomiasis, is transmitted by blood-sucking bugs. The disease can also be transmitted from mother to child during pregnancy and childbirth, via blood transfusion, organ transplantation and consumption of food or drink contaminated with the parasites. If untreated, it can cause irreversible damage to the heart and other vital organs. The interviewed immigrants constructed their knowledge about the disease primarily through stories of friends, neighbours, and relatives who suffered from the impacts of the disease, as well as from healthcare workers. The study explored barriers to accessing health services by people with Chagas disease such as fear of diagnosis, language competence, availability of hospital appointments and long waiting times (Silveira et al.).

The article by Agrawal et al. focuses on STH in 5 to 18-year-old schoolchildren in low- and middle-income countries. The study includes a systematic literature review and meta-analysis of disease prevalence in the Western Pacific, Africa, Western Pacific, Southeast Asia, Eastern Mediterranean and Europe. The prevalence of four groups of helminthiases caused by Ascaris roundworm (*Ascaris lumbricoides*), hookworm (*Ancylostoma duodenale, Ancylostoma ceylanicum*, and *Necator americanus*), whipworm (*Trichuris trichiura*) and pinworm (*Enterobius vermicularis*) was analysed. The study estimated that the overall pooled prevalence of helminthiases in schoolchildren was around 37%, with the highest prevalence in the Western Pacific region at around 50%. The roundworm *Ascaris lumbricoides* was the most prevalent helminth (24% of helminthiases). The study concluded that mass drug administration (MDA) of albendazole and improved water, sanitation, and hygiene (WASH) programmes are crucial for controlling STH (Agrawal et al.).

The article by Gwayi-Chore et al. analysed the networks of stakeholders who influence the delivery of both school-based and community-wide MDA for STH in Benin, India, and Malawi. The authors used social network analysis as part of the implementation research arm of a hybrid clinical trial, to systematically determine how network dynamics may impact implementation and scale-up of these programmes. The analysis demonstrated that across various administrative levels responsible for MDA planning and delivery, a wide range of stakeholders were involved, the majority of whom held positive attitude scores towards both programmes. Networks appeared to be stable in that no single individual exhibited high control over resource flow, though connectedness was poor overall due to minimal connectivity across administrative levels. These findings provide useful insights towards optimising effectiveness and efficiency of public health programs, specifically those in STH-endemic countries aiming to successfully interrupt STH transmission by transitioning from school-based to community-wide MDA.

The mini-review by Faust and Ray summarises publications on the accessibility to post-exposure treatment for rabies and snakebite, which each year take 179,000 human lives worldwide. The review highlighted the main challenges of timely access to treatment: under-reporting, distance to healthcare facilities, availability of treatments at nearby facilities, compliance with treatment, seeking help from traditional healers and cost of travel. Interventions to provide health care to bite victims may include: establishing a system where health professionals can report shortages of treatments at their facilities via mobile phones; providing a hotline service where bite victims can call and be directed to the nearest facility with available treatment; establishing volunteer-based motorcycle transportation for bite victims; training staff and raising awareness in local communities (Faust and Ray).

The article by Hounsome et al. reports the results of a modelling study for control of scabies in Ethiopia. Scabies is caused by the mite *Sarcoptes scabiei*, which lives under the skin, leading to intense itch and secondary skin infections. The transmission occurs by skin-to-skin contact with infected individuals and also via infested clothing and bedding. A decision-analytic model was developed to estimate the number of scabies cases and costs of two treatment strategies—ivermectin-based MDA and topical treatments with permethrin, benzyl benzoate, and sulphur cream. The model allows for changing population size, scabies prevalence, effectiveness of treatments, compliance with MDA and medication cost. The model can be used by decision-makers for planning scabies control programmes and integrating them with other MDA interventions (Hounsome et al.).

This collection of articles redirects attention from merely assessing the efficacy of NTD treatments to examining barriers to early diagnostics, access to NTD services and implementation of control programmes. Understanding how individuals and communities perceive NTDs and the factors that influence their decisions to seek medical help is essential for developing new interventions targeting NTDs (Ackley et al., Faust and Ray, Silveira et al.). Decision modelling emerges as a vital tool for policymakers involved in the planning and implementation of interventions for NTDs. The models can assist with estimating thresholds for MDA initiation and cessation and predict the outcomes and associated costs (Hounsome et al.). Social network analysis is another emerging approach that can help identify key stakeholders and understand the role of individuals, groups, and institutions in delivering community-based public health programs (Gwayi-Chore et al.). To achieve the ambitious targets the WHO set for NTDs by 2030, efforts should be made to integrate platforms to deliver NTD interventions and improve programmes' cost-effectiveness, coverage and geographical reach (Agrawal et al., Faust and Ray, Hounsome et al., 3).
